# A Seroepidemiological Study of Serogroup A Meningococcal Infection in the African Meningitis Belt

**DOI:** 10.1371/journal.pone.0147928

**Published:** 2016-02-12

**Authors:** Olivier Manigart, Caroline Trotter, Helen Findlow, Abraham Assefa, Wude Mihret, Tesfaye Moti Demisse, Biruk Yeshitela, Isaac Osei, Abraham Hodgson, Stephen Laryea Quaye, Samba Sow, Mamadou Coulibaly, Kanny Diallo, Awa Traore, Jean-Marc Collard, Rahamatou Moustapha Boukary, Oumarou Djermakoye, Ali Elhaji Mahamane, Jean-François Jusot, Cheikh Sokhna, Serge Alavo, Souleymane Doucoure, El Hadj Ba, Mariétou Dieng, Aldiouma Diallo, Doumagoum Moto Daugla, Babatunji Omotara, Daniel Chandramohan, Musa Hassan-King, Maria Nascimento, Arouna Woukeu, Ray Borrow, James M. Stuart, Brian Greenwood

**Affiliations:** 1 Faculty of Infectious Diseases, London School of Hygiene & Tropical Medicine, London, United Kingdom; 2 Medical Research Council Unit, Fajara, The Gambia; 3 Department of Veterinary Medicine, University of Cambridge, Cambridge, United Kingdom; 4 Public Health England Vaccine Evaluation Unit, Manchester, United Kingdom; 5 Armauer Hansen Research Institute, Addis Ababa, Ethiopia; 6 Navrongo Health Research Centre, Navrongo, Ghana; 7 Centre pour les Vaccins en Développement, Bamako, Mali; 8 Centre de Recherche Médicale et Sanitaire (CERMES), Niamey, Niger; 9 Institut de Recherche pour le Développement, Dakar, Senegal; 10 Centre de Support en Santé International (CSSI), N'Djamena, Chad; 11 Department of Community Medicine, University of Maiduguri, Maiduguri, Nigeria; Georgia Institute of Technology, UNITED STATES

## Abstract

The pattern of epidemic meningococcal disease in the African meningitis belt may be influenced by the background level of population immunity but this has been measured infrequently. A standardised enzyme-linked immunosorbent assay (ELISA) for measuring meningococcal serogroup A IgG antibodies was established at five centres within the meningitis belt. Antibody concentrations were then measured in 3930 individuals stratified by age and residence from six countries. Seroprevalence by age was used in a catalytic model to determine the force of infection. Meningococcal serogroup A IgG antibody concentrations were high in each country but showed heterogeneity across the meningitis belt. The geometric mean concentration (GMC) was highest in Ghana (9.09 μg/mL [95% CI 8.29, 9.97]) and lowest in Ethiopia (1.43 μg/mL [95% CI 1.31, 1.57]) on the margins of the belt. The force of infection was lowest in Ethiopia (λ = 0.028). Variables associated with a concentration above the putative protective level of 2 μg/mL were age, urban residence and a history of recent vaccination with a meningococcal vaccine. Prior to vaccination with the serogroup A meningococcal conjugate vaccine, meningococcal serogroup A IgG antibody concentrations were high across the African meningitis belt and yet the region remained susceptible to epidemics.

## Introduction

Epidemics of meningococcal disease have occurred at irregular intervals across the Sahelian and sub-Sahelian regions of Africa, the African meningitis belt, for over 100 years.[1] However, despite many years of research it is still not known why epidemics occur at a particular place at any specific time. An important factor is likely to be the background level of immunity of the population when faced with a potentially epidemic strain. It is known that protective immunity to *Neisseria meningitidis* can be induced by meningococcal carriage,[2] infection with other non-pathogenic *Neisseria* species, such as *N*. *lactamica*[3]and possibly by other bacteria.[4,5] There is also some evidence that background immunity may be impaired with infection by other bacteria that induce blocking antibodies.[6] The immune response to meningococcal polysaccharide and conjugate vaccines has been studied in the African meningitis belt[7–10] on several occasions but there have been few studies of population levels of antibody to *N*. *meningitidis* in the African meningitis belt.[11,12] Therefore, we have undertaken a study of community levels of serogroup-specific IgG antibody to *N*. *meningitidis* serogroup A (NmA) in six countries in the African meningitis belt before the introduction of the serogroup A conjugate vaccine, MenAfriVac™, to investigate heterogeneity in the level of exposure across the meningitis belt and to use age specific antibody titres to measure the force of infection.[13] To ensure that patterns of antibody could be compared across sites, we implemented standardised methods supported by careful quality control.

## Materials and Methods

### Study population

Cross-sectional meningococcal carriage surveys were conducted in seven countries across the meningitis belt during the period July 1^st^ 2010 to July 31^st^ 2012 as described previously.[14] Ethical approval for the study was obtained from the London School of Hygiene & Tropical Medicine and from an appropriate committee from each African centre. Written, informed consent for study participation was obtained from adults and for the children under their care. Written informed assent was also obtained from participants aged 12 years or more. Oral assent was obtained from younger children.

Subjects were selected randomly from within populations which were part of a routine demographic survey system (DHSS) or in which a census had been performed recently. The study population was recruited from urban and rural populations and stratified into four age groups: < 5 years, 5–14 years, 15–29 years and 30 years or older.

Subjects were asked if they had received a meningitis vaccine in the previous six months. Approximately a year before the survey, a vaccination campaign with an A + C polysaccharide vaccine had been conducted in the study area in Senegal and also in part of the urban study area in Niger.[15] None of the study populations had been vaccinated with MenAfriVac™ at the time of the survey.

Blood samples were collected from the first 100 subjects surveyed within each of the four age bands in both urban and rural study sites, giving an overall target of 800 samples per country. This target was achieved, or nearly achieved, except in Senegal where there was some resistance to the collection of blood samples. A 5 mL sample was collected, serum separated within six hours of collection and then stored at -20°C until assayed.

### Enzyme Linked Immuno-Sorbent Antibody (ELISA) assay

An internationally standardised ELISA, as used at the Vaccine Evaluation Unit (VEU), Public Health England, Manchester, UK was transferred to each of the MenAfriCar centres. Concentrations of IgG antibody against *N*. *meningitidis* serogroup A polysaccharide were obtained through a classical sandwich assay ELISA as described previously,[16] except that the standard reference serum CDC1992 was used as the quantification reference and that a monoclonal-PAN anti-human IgG Fc labelled with horseradish peroxidase (HRP)(Hybridoma Reagent Laboratory, Baltimore, MD) was used as conjugate. The lower limit of quantification (LLQ) of the meningococcal serogroup A ELISA was 0.19 μg/mL. Any value lower than the LLQ was assigned a value of 0.095 μg/mL for computational purposes.

### Standardisation of the assay and quality control

To ensure comparability of assay methods between centres, two training sessions were held in Manchester, UK and in Bamako, Mali at the start of the project. Subsequently, approximately 50 samples obtained during a pilot study conducted in each country were selected to allow cross-validation of the technique between each of the centres and the VEU, Manchester. After repeated testing of the 50 samples and adjustment of the technique to ensure that the results obtained fell within a defined range of the results obtained at the VEU, authorization was given to start testing the samples obtained during the cross-sectional survey. During the course of testing, monitoring of key values was performed by a resident scientist: two values representative of the standard curves (average of the duplicate values of the second higher concentration point and midpoint of the slope) as well as the calculated concentration of the local positive control were plotted routinely on Levey-Jennings charts. Regular review of these data was undertaken by the MenAfriCar laboratory manager and advice provided on adjustment of the technique when problems arose, for example detection of degradation in the anti-IgG conjugate used in the assay. It was not possible to complete cross-validation in Nigeria due to increasing insecurity and samples collected in Nigeria were tested in Mali. The laboratory in Chad did not reach the required standard in the validation assay to progress to testing of cross-sectional samples.

### Statistical methods

Pearson’s correlation coefficient (ρ) was used to compare the results produced at the VEU and by each centre during the validation exercise. The acceptance criterion for passing the cross-validation test was ρ greater than or equal to 0.9. In addition, Lin’s concordance coefficient of correlation, (ρC), which evaluates the degree to which pairs of observations fall on the 45° line through the origin and which provides a measure of both precision and accuracy of an assay was used.[17]

For analysis of the results from samples obtained during the cross-sectional surveys, geometric mean antibody concentrations (GMCs) were calculated and the percentage of samples reaching the putative protective threshold of ≥2 μg/mL,[18] together with 95% confidence intervals, was determined. Results by country were analysed graphically using reverse cumulative distribution plots. GMCs were compared by urban /rural residence and by sex in each country using a t-test. Risk factors for seropositivity (i.e. antibody concentration ≥2 μg/mL) were investigated using logistic regression. A multivariable logistic regression model was developed as follows: all variables with a p-value <0.1 in univariable analyses were included initially, then any variable with p-value <0.05 in the multivariable model was retained, with excluded variables re-entered one by one. If any of the re-entered variables had a p-value <0.05, they were retained in the final model. Because the survey was designed with the household as the primary sampling unit, and to account for potential household clustering, we used the survey commands in Stata (StataCorp, Texas).

Seroprevalence was stratified into yearly age groups and then analysed using a reverse catalytic modelling approach under a binomial sampling assumption, as described elsewhere.[13] Two key parameters were estimated using this approach (1) the seroconversion rate (SCR), i.e. the annual rate at which individuals change from seronegative to seropositive, also known as the force of infection (λ) and (2) the seroreversion rate, the annual rate at which seropositive individuals revert to a seronegative state (SRR or r). The catalytic model was fitted using a maximum likelihood approach. Analyses were repeated, excluding individuals who reported recent vaccination to estimate ‘natural’ immunity.

All analyses were performed using Stata v12.0.

### Ethics

The purpose and methods of the study were explained to community leaders at community meetings and through the media. Written, informed consent for obtaining a pharyngeal swab and a blood sample was obtained from adults and for the children under their care. Written informed assent was also obtained from participants aged 12 years or more. Oral assent was obtained from younger children. Consent and assent forms were translated into the relevant local language.

The study protocols, consent and assent forms were approved by the LSHTM Ethics Committee and by the ethics committees of each of the African partner institutions with the exception of Chad, which does not have a formal ethical committee, and where approval for the activities of the consortium was granted by a committee set up to oversee MenAfriCar studies by the Ministry of Health.

## Results

### Cross-validation

Although the cross-validation exercise required several rounds of testing, five centres finally achieved excellent results with Pearson correlation values (ρ) between 0.926 and 0.996 (Table 1). Final results obtained in Ghana are shown as an example in S1 Fig. Following the validation exercise, quality control of the results obtained on analysis of the cross-sectional survey samples was ensured by monitoring the key parameters of the standard curve as well as the local positive controls, as shown for Ethiopia in S2A Fig.

**Table 1:** Statistical analysis of a comparison of meningococcal serogroup A IgG concentrations obtained at the Vaccine Evaluation Unit (VEU), Public Health England and at five MenAfriCar centres.

Variable/ Country	Ethiopia	Ghana	Mali 1	Mali 2*	Niger	Senegal
Number of sera tested	50 (29 + 21)	49	50	29	50	60 (39 + 21)
Range of values tested (ug/ml)	0.095–133.38	0.34–133.38	0.43–67.42	0.095–3.42	0.095–79.36	0.28–133.38
Pearson’s correlation coefficient (ρ)	0.996	0.994	0.859	0.947	0.938	0.996
Lin’s concordance coefficient (ρc)	0.988	0.991	0.858	0.825	0.757	0.966
Slope	1.135	0.930	0.970	1.338	0.577	1.281
Intercept	-0.957	-0.372	0.010	0.159	0.966	-0.703

Footnote

* Two sets of validation samples were used for Mali because the correlation co-efficient from the first 50 samples did not reach the required threshold of 0.9. The second set of samples was selected from a set tested at the VEU which gave consistent results when tested in Manchester, UK.

### Meningococcal serogroup A IgG antibodies by country and by sub-group

**Fig 1:**
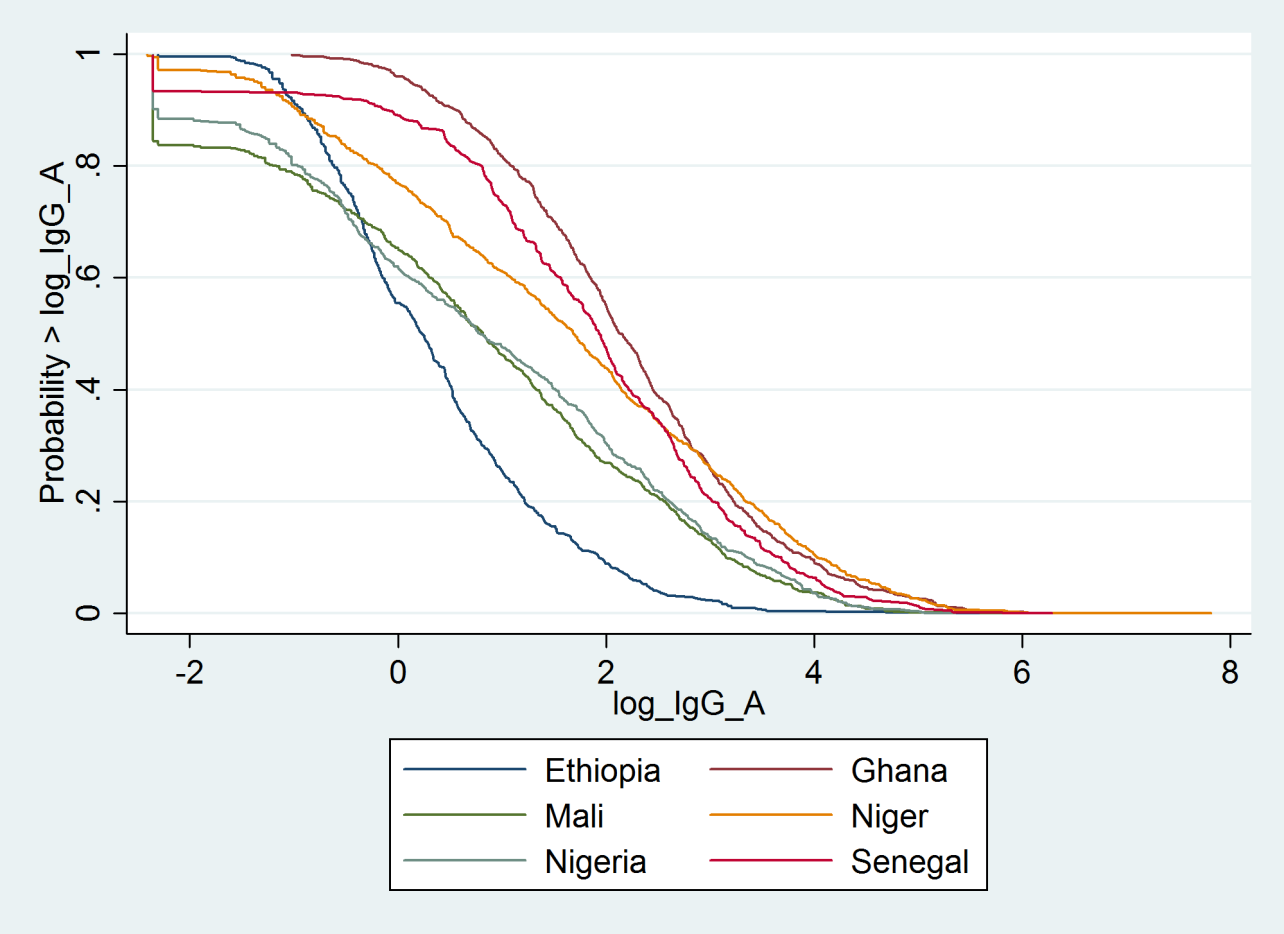
Reverse cumulative distribution curves of meningococcal serogroup A IgG antibodies by country.

Sera obtained from 3930 individuals in six African meningitis belt countries were tested. The prevalence of serogroup A specific IgG antibodies for each country is shown as a reverse cumulative plot in Fig 1. There were significant differences GMC by country with Ghana having the highest mean GMC and Ethiopia the lowest (Table 2).

**Table 2:** Geometric mean meningococcal serogroup A IgG antibody concentrations and seroprevalence by country.

A. All individuals			
**Country**	**Number**	**GMC (95% CI)**	**% ≥2ug/ml (95% CI)**
Ethiopia	619	1.43 (1.31, 1.57)	33.8% (30.0, 37.5%)
Ghana	765	9.09 (8.29, 9.97)	87.6% (85.2, 89.9%)
Mali	756	2.04 (1.77, 2.34)	52.1% (48.5, 55.7%)
Niger	826	4.98 (4.37, 5.66)	65.6% (62.3, 68.8%)
Nigeria	584	2.24 (1.91, 2.62)	52.1% (48.1, 56.2%)
Senegal	380	5.90 (5.00, 6.96)	81.1% (77.1, 85.0%)
B. Excluding individuals with a history of recent meningococcal vaccination			
**Country**	**Number**	**GMC (95% CI)**	**% ≥2ug/ml (95% CI)**
Ethiopia	617	1.43 (1.31, 1.57)	33.7% (30.0, 37.4%)
Ghana	650	8.70 (7.89, 9.59)	87.2% (84.6, 89.8%)
Mali	706	2.10 (1.82, 2.43)	52.7% (49.0, 56.4%)
Niger	757	5.24 (4.59, 5.99)	66.8% (63.5, 70.2%)
Nigeria	559	2.20 (1.88, 2.58)	52.0% (47.9, 56.2%)
Senegal	178	3.96 (3.05, 5.15)	74.7% (68.3, 81.1%)

The GMC increased with age in all countries with the exception of Senegal where there was a drop in GMC in those over 30 years of age (Table 3). This was less apparent when subjects with a history of recent vaccination were excluded. In four countries, no differences in GMCs by sex were observed (Ethiopia, Niger, Nigeria, Senegal, p>0.2 in each country); in Ghana and Senegal GMCs were higher in females compared to males (p = 0.0002 and p = 0.0203, respectively). A comparison was made in each country between GMCs according to whether the study site was urban or rural and by the sex of the participant. In five countries, NmA-specific IgG GMCs were higher in urban than in rural areas (p<0.0001 for Ethiopia, Ghana, Mali and Niger, p = 0.0012 for Senegal); in Nigeria, GMC was higher in the rural study site (p = 0.0123).

**Table 3:** Geometric mean meningococcal serogroup A IgG antibody concentrations (95% CI) by age and country.

A. All individuals				
**Country**	**Age group (years)**			
	**< 5**	**5–14**	**15–29**	**> = 30**
**Ethiopia**	0.62 (0.53, 0.72)	0.89 (0.78, 1.03)	1.86 (1.59, 2.17)	2.68 (2.30, 3.14)
**Ghana**	2.48 (1.92, 3.19)	6.25 (5.06, 7.72)	11.59 (9.97, 13.47)	12.06 (10.55, 13.80)
**Mali**	0.28 (0.13, 0.34)	1.89 (1.46, 2.46)	5.75 (4.67, 7.08)	5.86 (4.84, 7.10)
**Niger**	0.89 (0.72, 1.10)	3.39 (2.67, 4.31)	10.99 (8.90, 13.57)	14.36 (11.89, 17.35)
**Nigeria**	0.39 (0.30, 0.51)	1.00 (0.78, 1.29)	5.07 (3.95, 6.50)	8.88 (7.25, 10.88)
**Senegal**	1.84 (1.18, 2.87)	7.40 (5.46, 10.02)	11.95 (9.01, 15.87)	4.26, 7.07)
B. Excluding individuals with a history of recent meningococcal vaccination				
**Country**	**Age group (years)**			
	**< 5**	**5–14**	**15–29**	**> = 30**
**Ethiopia**	0.62 (0.53, 0.72)	0.89 (0.78, 1.03)	1.86 (1.59, 2.17)	2.68 (2.30, 3.14)
**Ghana**	2.41 (1.81, 3.22)	5.79 (4.64, 7.23)	11.36 (9.65, 13.38)	11.29 (9.81, 13.00)
**Mali**	0.26 (0.22, 0.32)	1.92 (1.46, 2.53)	5.67 (4.59, 7.00)	6.04 (4.98, 7.33)
**Niger**	0.94 (0.75, 1.19)	3.50 (2.72, 4.50)	10.89 (8.73, 13.57)	14.26 (11.77, 17.28)
**Nigeria**	0.39 (0.30, 0.51)	0.89 (0.70, 1.14)	4.88 (3.81, 6.26)	8.59 (7.02, 10.52)
**Senegal**	0.94 (0.41, 2.19)	3.68 (1.41, 9.60)	6.21 (3.28, 11.78)	5.10 (3.86, 6.74)

Factors associated with NmA-specific IgG concentrations above the putative protective threshold of 2μg/ml are shown in Table 4. The multivariable model included the following factors shown to be associated with a higher odds of seropositivity in addition to observed country-level differences: urban location, increasing age and reported receipt of a meningitis vaccine which is likely to have contained the serogroup A polysaccharide. Current carriage of meningococci or other *Neisseria* species were not associated with seropositivity in the multivariable model.

**Table 4:** Logistic regression analysis of factors associated with a meningococcal serogroup A IgG concentration ≥2ug/ml, a putative correlate of protection.

Variable	N	Crude Odds Ratio (95% CI)	Adjusted Odds Ratio (95% CI)
*Country*			
Ethiopia	619	0.27 (0.21, 0.33)	0.15 (0.11, 0.20)
Ghana	765	3.72 (2.83, 4.91)	2.79 (2.03, 3.85)
Mali	756	0.57 (0.46, 0.71)	0.49 (0.38, 0.64)
Niger (baseline)	826	1.0	1.0
Nigeria	583	0.57 (0.46, 0.71)	0.44 (0.34, 0.58)
Senegal	380	2.24 (1.65, 3.00)	2.02 (1.31, 3.12)
*Age group*			
0 to 4 years	746	0.09 (0.07, 0.11)	0.06 (0.05, 0.08)
5 to 14 years	957	0.32 (0.27, 0.39)	0.26 (0.21, 0.32)
15 to 29 years (baseline)	1023	1.0	1.0
30+ years	1203	1.59 (1.29, 1.96)	1.68 (1.33, 2.12)
*Sex*			
Female	2241	1.0	Not included
Male	1668	0.82 (0.72, 0.93)	
Not known	20		
Urban	1871	1.0	1.0
Rural	2058	0.64 (0.56, 0.73)	0.54 (0.45, 0.64)
*Recently vaccinated with a meningitis vaccine*			
No	3459	1.0	1.0
Yes	463	2.09 (1.65, 2.66)	1.71 (1.23, 2.38)
*Meningococcal carrier*			
No	3771	1.0	Not included
Yes	158	0.94 (0.67, 1.31)	
*Carrier of other* *Neisseria* *species*			
No	3706	1.0	Not included
Yes	224	0.40 (0.30, 0.53)	
Crowded living conditions*			
No	1438	1.0	Not included
Yes	2484	0.70 (0.60, 0.81)	

* crowding was defined as > = 2 people per room as used previously[27]

**Fig 2:**
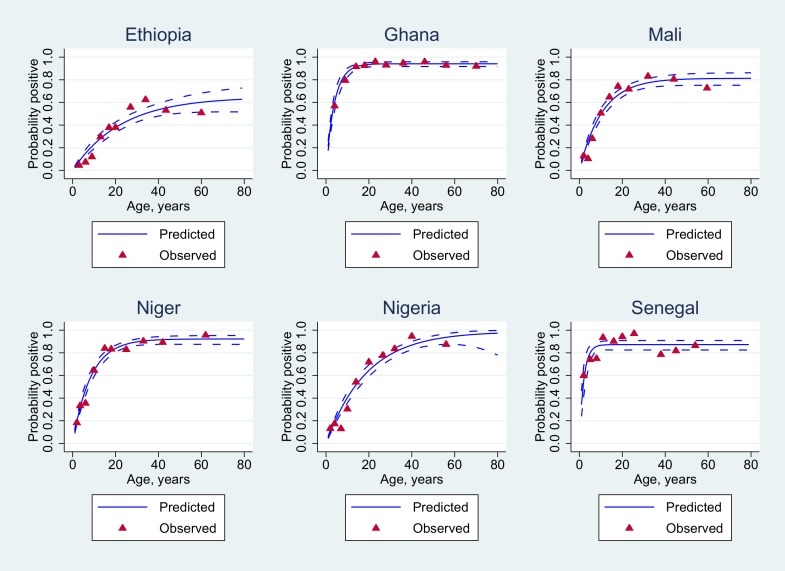
Statistical analysis of seropositivity data for all individuals. Age-adjusted seroprevalence (blue solid lines) using appropriate reversible catalytic models. The observed seroprevalences (red-filled triangles) were pooled according to the 10%-centiles of the underlying age distribution.

**Fig 3:**
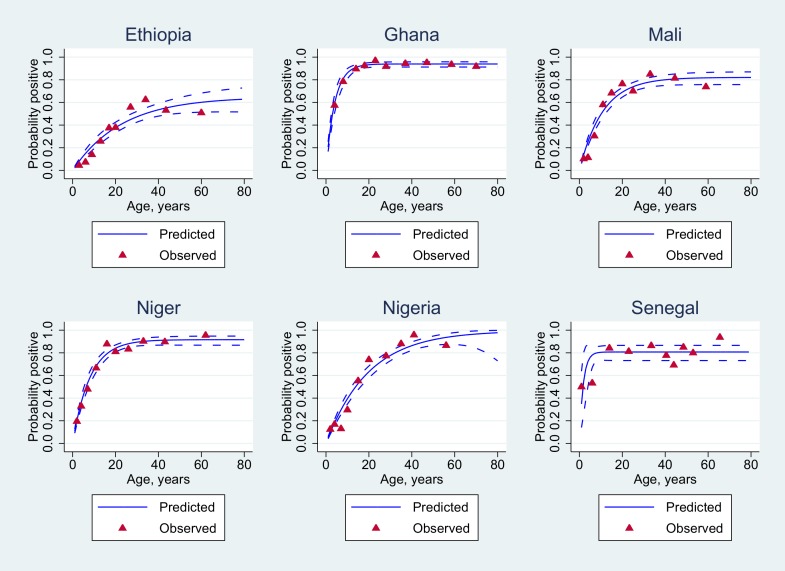
Statistical analysis of seropositivity data excluding vaccinated individuals. Age-adjusted seroprevalence (blue solid lines) using appropriate reversible catalytic models. The observed seroprevalences (red-filled triangles) were pooled according to the 10%-centiles of the underlying age distribution.

The influence of age on the putative protective threshold of 2 μg/mL is further shown by country in Figs 2 and 3.

### Force of infection

The force of infection was calculated based on the age prevalence of seropositivity as described above. Unexpectedly, this was highest in Senegal, even when subjects with a recent history of vaccination were excluded, followed by Ghana and Niger; Ethiopia had the slowest seroconversion rate (Table 5).

**Table 5:** Estimates of the annual force of infection (λ) and seroreversion (r) by country.

A. All individuals		
**Country**	**λ (95% CI)**	**r (95% CI)**
Ethiopia	0.028 (0.022, 0.036)	0.015 (0.007, 0.033)
Ghana	0.240 (0.192, 0.299)	0.015 (0.009, 0.024)
Mali	0.077 (0.064, 0.091)	0.017 (0.011, 0.027)
Niger	0.110(0.093, 0.130)	0.009 (0.005, 0.018)
Nigeria	0.052 (0.044, 0.062)	0.005 (0.000, 0.485)
Senegal	0.434 (0.282, 0.670)	.033, 0.124)
B. Excluding individuals with a history of recent meningococcal vaccination		
**Country**	**λ (95% CI)**	**r (95% CI)**
Ethiopia	0.028 (0.022, 0.036)	0.015 (0.007, 0.333)
Ghana	0.233 (0.183, 0.295)	0.015 (0.008, 0.025)
Mali	0.075 (0.063, 0.090)	0.016 (0.010, 0.027)
Niger	0.114 (0.095, 0.137)	0.010 (0.005, 0.020)
Nigeria	0.050 (0.042, 0.060)	0.0002 (0, 1)
Senegal	0.457 (0.160, 1.30)	0.109 (0.031, 0.380)

## Discussion

Although a number of studies of meningococcal serology have been performed previously in countries of the African meningitis belt, most of these have been undertaken in the context of evaluation of the response to vaccination[7–10] or in disease survivors.[19] Comparisons between the results obtained in individual studies can be difficult when standardised methods are not used. Therefore, for this comparative study, substantial efforts were made to standardise the ELISA technique used at each of the collaborating centres with support from the Vaccine Evaluation Unit at PHE, Manchester, UK. This proved more challenging than anticipated and satisfactory results were not obtained at one out of six centres prior to the end of the project. Problems encountered included the short shelf-life of some of the reagents required, difficulties in clearing reagents through customs and difficulties in shipping frozen samples to the UK for validation. However, despite these challenges, excellent results were eventually obtained at five centres using Pearson coefficient of correlation (r>0.92) and satisfactory ones using Lin’s concordance coefficient of correlation, allowing adjustment of some issues which were not identified using the correlation coefficient alone.

Overall, serogroup A meningococcal IgG antibody concentrations were high in the African populations investigated, with the highest GMCs being obtained in countries in the centre of the meningitis belt. This has been noted previously.[10,11] This was the case despite the fact that there was little circulation of the serogroup A meningococcus in the African meningitis belt at the time of the study.[20] These high antibody concentrations probably reflect prior exposure to the serogroup A meningococcus and other cross-reactive bacteria such as *Bacillus pumilis* [5,21] and *Escherichia co*li capsule types K51 or K93.[22] Similarly high serogroup A meningococcal IgG antibody concentrations have been found In industrialised countries where there is no group A disease and no evidence of carriage. For example, in England and Wales a GMC of 4.75 μg/mL was found in the general population with 87% of individuals with antibody levels ≥2 μg/mL,[23] an observation which supports the role for cross-reactive bacteria in inducing antibodies against the serogroup A meningococcus.

None of the populations in the MenAfriCar cross-sectional surveys had been vaccinated previously with MenAfriVac™ but meningococcal vaccines containing A polysaccharide have been used quite extensively in some of the study countries in the past. Subjects were asked whether they had received a meningitis vaccine in the previous six months and if this was the case, they are likely to have been vaccinated with a vaccine containing serogroup A polysaccharide. Analyses of antibody distribution by country and by age were undertaken excluding these subjects but this had little impact on the pattern of results. However, earlier vaccination campaigns may have resulted in some persistent antibody. Prior vaccination may be the reason for the rapid seroconversion rate in the Senegalese population where a mass vaccination campaign with an A + C polysaccharide vaccine had been undertaken in 2010, about 12 months prior to the study and also for the reason why the GMC was lower in subjects over 30 years of age in Senegal than in younger subjects who would not have been targeted in the immunisation campaign. Thus, the antibody distribution seen in each country may reflect a combination of responses induced by both natural exposure and vaccination.

An Ig meningococcal serogroup A polysaccharide antibody concentration of > 2 μg/mL as measured by radioimmunoassay has been suggested as a correlate of protection against serogroup A meningococcal disease based on the results of a trial of a meningococcal polysaccharide vaccine conducted in Finland in the 1970s.[18] However, it seems unlikely that this is the case in countries of the meningitis belt where a high proportion of the population has antibody concentrations above this value and yet, until the introduction of MenAfriVac™, the region remained peculiarly susceptible to large serogroup A epidemics. It is likely that much of the antibody detected by ELISA is non-functional, perhaps because it is induced by cross-reacting bacteria. Bactericidal antibodies are the accepted correlate of protection for meningococcal disease [24] and measurement of serogroup A serum bactericidal antibodies may give a better reflection of the background level of immunity of a community. However, these are technically more difficult to perform reliably than the ELISA, and were only undertaken at two of the centres that participated in this study before and after the introduction of MenAfriVac™. These results, together with studies of the correlates of protection against invasive meningococcal disease and meningococcal pharyngeal carriage will be reported subsequently.

In this study, we have shown that serology can be used to show differences in exposure to meningococcal infection between countries and age groups and we have shown how age dependent variations in seropositivity can be used to measure the force of infection. The force of infection was generally highest in countries in the centre of the meningitis belt, with the exception of Senegal where the situation may have been confounded by vaccination, and lowest in Ethiopia on the margin of the belt. Measurement of seroconversion by age has proved to be a valuable approach to study of the epidemiology of malaria and of the impact of control interventions on this infection [25,26] and, as shown here, is valuable for the study of other infectious diseases. Further study of antibody kinetics in the African meningitis belt is important for understanding the epidemiology of meningococcal infection and monitoring control measures including widespread deployment of conjugate vaccines.

## Supporting Information

S1 Fig (DOCX)

S2 Fig (DOCX)
