# Correction: A Seroepidemiological Study of Serogroup A Meningococcal Infection in the African Meningitis Belt

**DOI:** 10.1371/journal.pone.0158938

**Published:** 2016-07-05

**Authors:** Olivier Manigart, Caroline Trotter, Helen Findlow, Abraham Aseffa, Wude Mihret, Tesfaye Moti Demisse, Biruk Yeshitela, Isaac Osei, Abraham Hodgson, Stephen Laryea Quaye, Samba Sow, Mamadou Coulibaly, Kanny Diallo, Awa Traore, Jean-Marc Collard, Rahamatou Moustapha Boukary, Oumarou Djermakoye, Ali Elhaji Mahamane, Jean-François Jusot, Cheikh Sokhna, Serge Alavo, Souleymane Doucoure, El Hadj Ba, Mariétou Dieng, Aldiouma Diallo, Doumagoum Moto Daugla, Babatunji Omotara, Daniel Chandramohan, Musa Hassan-King, Maria Nascimento, Arouna Woukeu, Ray Borrow, James M. Stuart, Brian Greenwood

The fourth author's name is spelled incorrectly. The correct name is: Abraham Aseffa. The correct citation is: Manigart O, Trotter C, Findlow H, Aseffa A, Mihret W, Moti Demisse T, et al. (2016) A Seroepidemiological Study of Serogroup A Meningococcal Infection in the African Meningitis Belt. PLoS ONE 11(2): e0147928. doi:10.1371/journal.pone.0147928
