# Supplementary material for: A Seroepidemiological Study of Serogroup A Meningococcal Infection in the African Meningitis Belt
Source: PLoS One. 2016 Feb 12;11(2):e0147928. doi: 10.1371/journal.pone.0147928 (PMC4752490; doi:10.1371/journal.pone.0147928)

**S1 Fig**

A comparison of meningococcal serogroup IgG concentration results produced at the Vaccine Evaluation Unit, Public Health England, Manchester, UK and at the Navrongo Health Research Institute, Ghana, during the cross-validation.


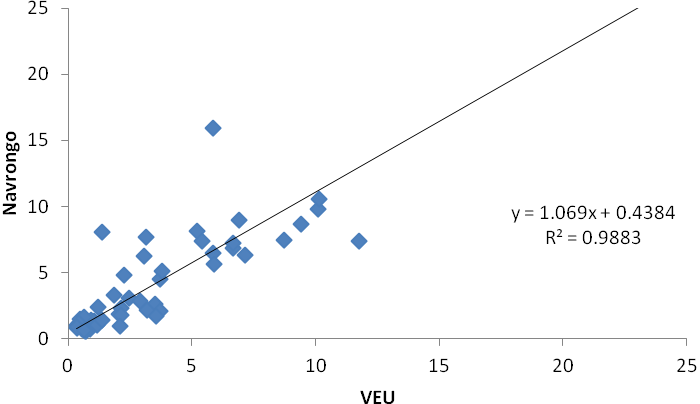

Supplement: S1 Fig — (DOCX) [file pone.0147928.s001.docx]
