# Supplementary material for: A Seroepidemiological Study of Serogroup A Meningococcal Infection in the African Meningitis Belt
Source: PLoS One. 2016 Feb 12;11(2):e0147928. doi: 10.1371/journal.pone.0147928 (PMC4752490; doi:10.1371/journal.pone.0147928)

**S2 Fig.**

Parameters measured during testing of the cross-sectional samples using results from Ethiopia as an example. Figure A - local positive control (QC), Figure B - average between duplicate of the second highest concentration of the standard curve (OD) and Figure C - angular coefficient of the linear part of the curve (Mid-point). The green asymptotes represent the upper accepted range and the red ones the lower. Asymptotes values for OD and mid-point were the same as the ones used at the reference laboratory, the Vaccine Evaluation Unit, Public Health England, Manchester.

**Figure A.**


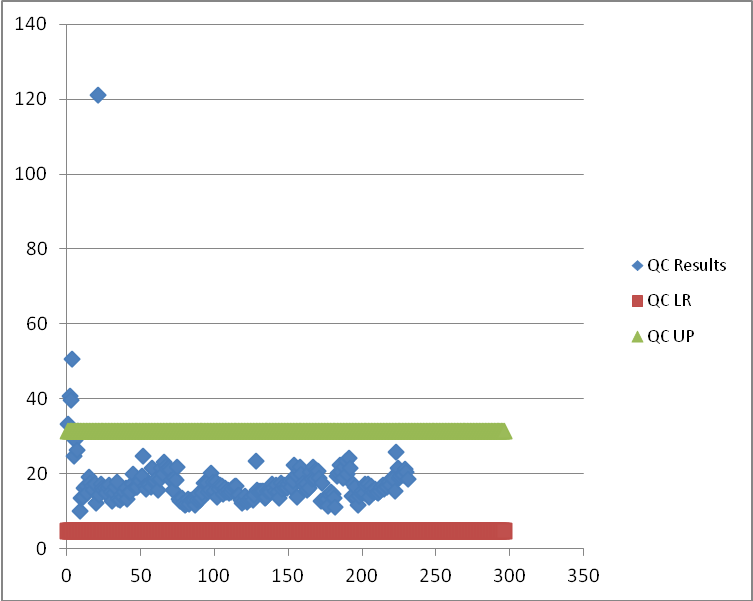


**Figure B.**


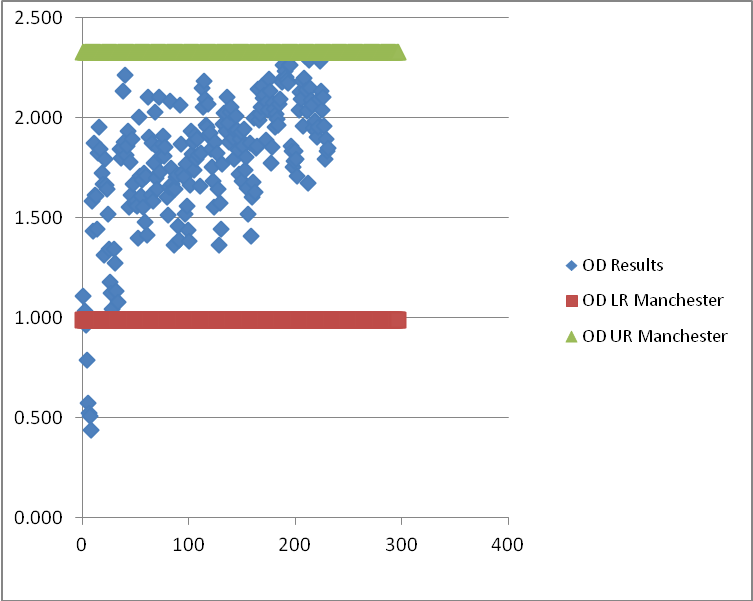


**Figure C.**


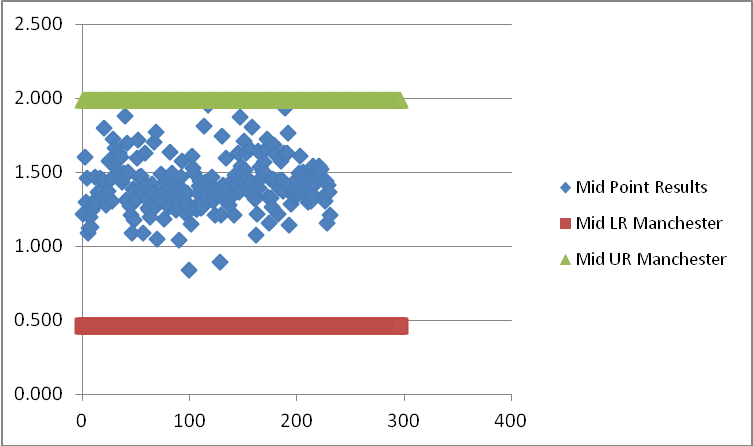

Supplement: S2 Fig — (DOCX) [file pone.0147928.s002.docx]
